# Molecular phylogeny, divergence time estimates and historical biogeography within one of the world's largest monocot genera

**DOI:** 10.1093/aobpla/plw041

**Published:** 2016-08-08

**Authors:** Qin-Qin Li, Song-Dong Zhou, De-Qing Huang, Xing-Jin He, Xian-Qin Wei

**Affiliations:** 1 Key Laboratory of Bio-Resources and Eco-Environment, MOE, College of Life Sciences, Sichuan University, Chengdu 610064, China Sichuan; 2 College of Life Science and Technology, Inner Mongolia Normal University, Hohhot 010022, China Inner Mongolia

**Keywords:** Allium, anguinum, divergence time, historical biogeography, hybridization/introgression, incomplete lineage sorting, phylogeny, radiation

## Abstract

A primary aim of historical biogeography is to identify the causal factors or processes that have shaped the composition and distribution of biotas over time. Another is to infer the evolution of geographic ranges of species and clades in a phylogenetic context. To this end, historical biogeography addresses important questions such as: Where were ancestors distributed? Where did lineages originate? Which processes cause geographic ranges to evolve through time? *Allium* subgenus *Anguinum* comprises approximately twelve taxa with a disjunct distribution in the high mountains from south-western Europe to eastern Asia and in northeastern North America. Although both the systematic position and the geographical limits of *Anguinum* have been identified, to date no molecular systematic study has been performed utilizing a comprehensive sampling of these species. With an emphasis on the *Anguinum* eastern Asian geographical group, the goals of the present study were: (i) to infer species-level phylogenetic relationships within *Anguinum*, (ii) to assess molecular divergence and estimated the times of the major splits in *Anguinum* and (iii) to trace the biogeographic history of the subgenus. Four DNA sequences (ITS, *matK*, *trnH-psbA*, *rps16*) were used to reconstruct the phylogeny of *Allium* subgen. *Anguinum*. *RbcL* sequences were used to estimate divergences time for *Allium*, and sequences of ITS were used to estimate the divergence times for *Anguinum* and its main lineages and to provide implications for the evolutionary history of the subgenus. Phylogenetic analyses for all *Allium* corroborate that *Anguinum* is monophyletic and indicate that *Anguinum* is composed of two sister groups: one with a Eurasian–American distribution, and the other restricted to eastern Asia. In the eastern Asian geographical group, incongruence between gene trees and morphology-based taxonomies was recovered as was incongruence between data from plastid and nuclear sequences. This incongruence is likely due to the combined effects of a recent radiation, incomplete lineage sorting, and hybridization/introgression. Divergence time estimates suggest that the crown group of *Anguinum* originated during the late Miocene (ca. 7.16 Mya) and then diverged and dispersed. Biogeographic analyses using statistical dispersal–vicariance analysis (S-DIVA) and a likelihood method support an eastern Asia origin of *Anguinum*. It is inferred that in the late Pliocene/Early Pleistocene, with cooling climates and the uplift of the Himalayas and Hengduan Mountains, the ancestor of the eastern Asian alliance clade underwent a very recent radiation.

## Abstract (precis)

*Allium* subgenus *Anguinum* is composed of two sister groups. In the eastern Asian geographical group, incongruence between gene trees and morphology-based taxonomies was recovered as was incongruence between data from plastid and nuclear sequences. This incongruence is likely due to the combined effects of a recent radiation, incomplete lineage sorting, and hybridization/introgression. The crown group of *Anguinum* originated during the late Miocene, and eastern Asia was the ancestral area for *Anguinum*. It is inferred that in the late Pliocene/Early Pleistocene, with cooling climates and the uplift of the Himalayas and Hengduan Mountains, the ancestor of the eastern Asian alliance clade underwent a very recent radiation.

## Introduction

A primary aim of historical biogeography is to identify the causal factors or processes that have shaped the composition and distribution of biotas over time (Sanmartín 2007). Another major focus is to infer the evolution of geographic ranges of species and clades in a phylogenetic context (Ree and Smith 2008). To this end, many questions about the historical distributions of species may be of interest, such as: Where were ancestors distributed? Where did lineages originate? Which processes (e.g., vicariance or dispersal) cause geographic ranges to evolve through time? (Ree and Smith 2008; Xie *et al.* 2009). Many studies have addressed these biogeographic questions using phylogenetic analyses, molecular dating, and reconstruction of ancestral geographic ranges (e.g., Sytsma *et al.* 2004; Nie *et al.* 2006; Bell 2007; Sanmartín *et al.* 2008; Xie *et al.* 2009).

A brief introduction of the paleontological history of the Northern Hemisphere since the late Cretaceous helps to understand the biogeographic history of plant biota. Overall, the Earth’s climate became cooler through the Tertiary (Zachos *et al.* 2001), and the climate cooled gently from 50 to 35 million years (Myr) ago, then fluctuated until 15 Myr ago, after which the climate cooled progressively, culminating in the Quaternary (2–0 Myr ago) glaciations (Milne and Abbott 2002). Cooling climates in the latter part of the Tertiary forced the boreotropical flora retreat southwards to large refugial regions that preserved the warm wet climate that they needed. These refugia include eastern Asia, south-eastern Europe, eastern and western North America, and western Asia, and the ﬂoras concerned are termed Tertiary relict ﬂoras (Tiffney *1985a*,*b*; Wen 1999; Milne and Abbott 2002). Over the 2 million years of the Quaternary, these data indicate considerable and continuous climatic variation (Mitchell 1976; Smiley *et al.* 1991). Up to 24 glacial events of about 50–100 000 years each have occurred (van Donk 1976). The climatic oscillations of the Quaternary resulted in repeated drastic environmental changes that profoundly shaped the current distributions and genetic structures of many plant species in temperate zones of the Northern Hemisphere (Hewitt 1996, 2000, 2004). When the Tertiary period began, North America and Eurasia were each separated into western and eastern portions by epicontinental seaways (Tiffney 1985*a*; Tiffney and Manchester 2001). At the same time western North America was connected to East Asia across the Bering land bridge (BLB), while eastern North America was connected via Greenland to north-eastern Europe—a connection known as the North Atlantic land bridge (NALB; Tiffney 1985*b*). Different perspectives exist for the timing of the break up of the NALB (Tiffney 1985 *a*, *b*; Tiffney 2000; Wen 2000; Tiffney and Manchester 2001; Milne and Abbott 2002; Milne 2006), but it is thought that the NALB was available for plant exchanges from the early Tertiary to possibly as late as 15 Myr ago (Milne and Abbott 2002). It is known that the BLB connected eastern Asia and western North America at one time or another throughout the late Cretaceous and the Tertiary and was available for ﬂoristic exchanges until ca. 3.5 millions of years (Ma; Tiffney 1985 *a*, *b*; Wen 1999; Gladenkov *et al.* 2002; Milne 2006).

The genus *Allium* comprises about 920 species (Seregin *et al.* 2015), making it one of the largest monocotyledonous genera. *Allium* is a member of order Asparagales, family Amaryllidaceae, subfamily Allioideae (Fay and Chase 1996; APG III 2009; Chase *et al.* 2009). After Fay and Chase (1996), Friesen *et al.* (2000) and Chase *et al.* (2009), *Allium* (including *Caloscordum* Herb., *Milula* Prain and *Nectaroscordum* Lindl.) is the only genus in tribe Allieae. Previous molecular data suggested that *Allium* evolution proceeded in three separate evolutionary lines; subgenus *Anguinum* is a member of the second evolutionary line (Fritsch 2001; Fritsch and Friesen 2002; Friesen *et al.* 2006; Li *et al.* 2010; Choi *et al.* 2012). *Anguinum* contains approximately twelve taxa (nine species and three varieties) with a disjunct distribution in the high mountains from south-western Europe to eastern Asia and in northeastern North America (Fritsch and Friesen 2002). It is characterized by specific root anatomical characters (Fritsch 1992*a*), leaf and bulb structure (Pastor and Valdes 1985), hypogeal seed germination and *A. victorialis*-type seedlings (Druselmann 1992), uniovulate locules (Hanelt, 1992), narrow, branched and lengthwise-twisted septal nectarines (Fritsch 1992*b*) and a short vegetative period with the adaptation of the light regime under deciduous forest conditions (Pistrick 1992; Li *et al.* 2010). Unlike other *Allium* lineages, the seed testa sculpturing is very simple among species of *Anguinum* (Kruse 1984, 1988). Species of *Anguinum* also share similar metaphase chromosomes and the basic chromosome number *x* = 8 and all reported karyotypes are 2A type (Jing *et al.* 1999). Based on the consistency of its morphological, anatomical and cytological characteristics, it is a rather distinct and specialized group (Li *et al.* 2010). Previous molecular studies indicated that *Anguinum* is monophyletic and shares a more recent common ancestor with *Vvedenskya*, *Porphyroprason* and *Melanocrommyum* and is the sister group to *Caloscordum* (Friesen *et al.* 2006; Li *et al.* 2010). According to Friesen *et al.* (2006) and Li *et al.* (2010), two sister groups comprise this subgenus: one with a Eurasian–American distribution, and the other restricted to the Hengduan Mountains and adjacent areas.

Although both the systematic position and the geographical limits of *Anguinum* have been identified, to date no molecular systematic study has been performed utilizing a comprehensive sampling of these species. Thus, in order to better understand the phylogeny and historical biogeography of *Anguinum*, an extended population sampling of *Anguinum* species endemic to eastern Asia was incorporated in this study. With an emphasis on the *Anguinum* eastern Asian geographical group, the goals of the present study were: (i) to infer species-level phylogenetic relationships within *Anguinum* using four molecular markers ITS, *matK*, *trnH-psbA*, and *rps16*, (ii) to assess molecular divergence and estimated the times of the major splits in *Anguinum* and related these divergence times with external factors that might have contributed to the diversification of the subgenus and (iii) to trace the biogeographic history of the subgenus by provided a time frame to the biogeographic studies.

## Methods

### Taxon sampling

Our sampling strategy was designed to cover those taxonomic and geographic *Anguinum* groups that were underrepresented in previous analyses, especially from eastern Asia, and to build on previous studies (Friesen *et al.* 2006; Li *et al.* 2010). Fifty samples (ITS sequences for four samples downloaded in Genbank) representing twelve taxa of *Anguinum* with a focus on the eastern Asian geographical group, were sampled as the ingroup for phylogenetic reconstructions. Two species from subgenus *Caloscordum* (*A. neriniflorum*, *A. tubiflorum*) were designated as outgroups according to previous studies (Fritsch and Friesen 2002; Friesen *et al.* 2006; Nguyen *et al.* 2008; Li *et al.* 2010). GenBank accession numbers and voucher details referred to the above-mentioned taxa are given in [**see Supporting Information, Appendix S1**].

There are no known fossils of *Anguinum* or even *Allium*, so we first intend to estimate the divergence time of *Allium*, based on a relatively broad analysis of *rbcL* sequences of *Allium* (i.e., representatives almost in every subgenus of *Allium*) together with other samples from the order Asparagales and other monocots. According to previous systematic studies (e.g. Fay and Chase 1996; Vinnersten and Bremer 2001; Davis *et al.* 2004; Tamura *et al.* 2004; Leebens-Mack *et al.* 2005; Müller *et al.* 2006; Meng *et al.* 2008; Kim *et al.* 2010) and our preliminary phylogenetic analysis for 255 sequences of the groups of monocots, 152 sequences were chosen covering all orders and as many families as possible referred to the present study, in which 32 sequences (26 sequences were generated in this study) representing *Allium* spp. [**see Supporting Information, Appendix S2**]. To infer divergence times within *Anguinum*, 144 ITS sequences (34 sequences were generated in this study) that represented *Anguinum* and other subgenera and sections in *Allium*, plus two sequences of *Nothoscordum gracile* (family Amaryllidaceae, subfamily Allioideae, tribe Gilliesieae) and *Tulbaghia violacea* (family Amaryllidaceae, subfamily Allioideae, tribe Tulbaghieae) were used, and the selection of *Anguinum* taxa is based on haplotype analysis, and sequences belong to the same haplotype form the same taxa are removed (except *A. nanodes*) [**see Supporting Information, Appendix S3**].

### DNA extraction, amplification and sequencing

Genomic DNA was extracted from silica gel-dried or fresh leaves using the method of Doyle and Doyle (1987). Primers ITS4 and ITS5 (White *et al.* 1990) were used to amplify the ITS region followed the protocol of Li *et al.* (2010). The *rps16* intron was amplified with primers rpsF and rpsR2 (Oxelman *et al.* 1997) in accordance with the protocol of Marazzi *et al.* (2006). The intergenic spacer *trnH*-*psbA* was amplified using the primers trnH (GUG) F and psbAR (Hamilton 1999). The PCR programme was as follows: 94 °C for 4 min; 30 cycles of 94 °C for 30 s, 52 °C for 30 s, 72 °C for 1 min; and 72 °C for 7 min. The *rbcL* was amplified with primers rbcL N' and DBRBAS2 (Terachi *et al.* 1987) for 26 sequences. The *matK* was amplified with primers 3F_KIM and IR_KIM (Kim, unpublished). Their PCR parameters were same as follows: 94 °C for 4 min; 30 cycles of 94 °C for 1 min, 52 °C for 1 min, 72 °C for 1 min 30 s; and 72 °C for 10 min. PCR products were separated using 1.5 % (w/v) agarose TAE gel and purified using Wizard PCR preps DNA Purification System (Promega, Madison, WI, USA) following the manufacturer’s instructions. The purified PCR products were sequenced in an ABI 310 Genetic Analyzer (Applied Biosystems Inc.) using the PCR primers.

### Sequence comparisons and phylogenetic analyses

Forward and reverse sequences were assembled and edited with SeqMan (DNAstar package; DNAStar Inc., Madison, WI, USA). DNA sequences were initially aligned using the default pairwise and multiple alignment parameters in Clustal X (Jeanmougin *et al.* 1998) and then rechecked and adjusted manually as necessary using MEGA4 (Tamura *et al.* 2007). Gaps were positioned to minimize nucleotide mismatches and treated as missing data in phylogenetic analyses. Phylogenetic analyses were conducted by employing maximum-parsimony (MP) criteria and Bayesian inference (BI), using the programs PAUP* version 4.0b10 (Swofford 2003) and MrBayes version 3.1.2 (Ronquist and Huelsenbeck 2003), respectively. For MP, heuristic searches were carried with 1000 random addition sequence replicates. One tree was saved at each step during stepwise addition, and tree-bisection-reconnection (TBR) was used to swap branches. All characters were unordered and equally weighted. Gaps were treated as missing data. Bootstrap values were calculated from 6000 000 replicate analyses using ‘fast’ stepwise-addition of taxa and only those values compatible with the majority-rule consensus tree were recorded. Prior to a Bayesian analysis, MrModeltest version 2.2 (Nylander 2004) was used to select a best-fit model of nucleotide substitution under the Akaike infomation criterion. The Bayesian Markov chain Monte Carlo (MCMC) settings consisted of four independent runs with four chains each (one cold chain and three incrementally heated chains) for 6 million generations starting from random trees and sampling one of every 100 generations, by using default priors and estimating all parameters during the analysis. The first 25 % of the trees were discarded as burn-in. A 50 % majority-rule consensus tree of the remaining trees was produced.

The incongruence length difference (ILD) test of ITS vs. the combined chloroplast sequences (*rps16*, *matK*, and *trnH-psbA*) was carried out in PAUP* (Farris *et al.* 1994) to assess potential conflicts between the phylogenetic signal from different genomes. This test was implemented with 100 partition-homogeneity test replicates, using a heuristic search option with the simple addition of taxa, TBR branch swapping and MaxTrees set to 1000.

### Divergence time estimation

Since the strict molecular clock model was rejected for *rbcL* with *P <* 0.05 in Likelihood ratio tests (LRTs; Felsenstein 1988), divergences time of *Allium* was estimated using a Bayesian approach with an uncorrelated lognormal relaxed molecular clock model (Drummond *et al.* 2006), as implemented in the program BEAST v1.5.2 (Drummond and Rambaut 2007). An advantage of BEAST is its ability to estimate the topology and divergence times simultaneously. The BEAST analysis was run for 5 ×10^7^ generations with parameters sampled every 1000 generations and used the GTR + I+G substitution model selected by MrModeltest and randomly generated Starting Tree and the Yule tree prior. The fossil record of monocots is comparatively poor primarily due to problems of preservation (Herendeen and Crane 1995; Crepet, Nixon and Gandolfo 2004). A few fossils of the Asparagales have been reported from the late Eocene (Couper 1960; Muller 1981; Herendeen and Crane 1995) and they all may be too young to calibrate the crown clade of the order (Wikström *et al.* 2001; Janssen and Bremer 2004). Based on five molecular markers, Magallón and Castillo (2009) estimated the age of the crown Asparagales as 124.95 million years ago (Mya) for relaxed datings. We therefore used this age as the calibration point for the crown group Asparagales node, with a normally distributed standard deviation of 0.35. Based on eight reference fossils, Bremer (2000) estimated the split between *Acorus* and all other monocots to be more than 134 Myr old. Using a calibration point outside the monocots (within the eudicot order Fagales), Wikström *et al.* (2001) produced an age for extant (crown group node) monocots of 127–141 Mya. These two estimates fall into the range of ages estimated by Sanderson and Doyle (2001) and are in fairly close agreement with Good-Avila *et al.* (2006) who estimated the crown age of monocots to be 132 Myr old. As Chase (2004) wrote, we can draw some generalities about the ages of monocots from these studies. Thus, following Janssen and Bremer (2004) and Meng *et al.* (2008), we calibrated the crown age of monocots as 134 Mya, with a normally distributed standard deviation of 4.25. We also set the crown age of Amaryllidaceae at 87 Mya based on Janssen and Bremer (2004), with a normally distributed standard deviation of 0.01. The results were evaluated by the Tracer program 1.4 (Rambaut and Drummond 2007) and the first 10 % of the generations were discarded as burn-in. Samples from the posterior were summarized on the maximum clade credibility tree using TreeAnnotator version 1.5.2. (Drummond and Rambaut 2007) and the tree was visualized using FigTree ver. 1.3.1 (Rambaut 2009). The divergence times are given as the mean node heights and the 95 % highest posterior density (HPD) intervals in millions of years (Ma). According to Magallón and Castillo (2009), we rooted the tree with four species (*Ascarina swamyana*, *Chloranthus nervosus*, *Hedyosmum arborescens* and *Sarcandra chloranthoides*) from order Chloranthales.

Because the likelihood ratio tests rejected the molecular clock for the data (*P <* 0.05), divergence times within *Anguinum* were estimated using the same methods stated above and the GTR + I+G substitution model was selected by MrModeltest. The crown age of *Allium* was set to 34.26 Mya based on previous analysis, with a normally distributed standard deviation of 0.1. According to previous phylogenetic analyses (Fay and Chase 1996; Mes *et al.* 1997; Fay *et al.* 2000; Friesen *et al.* 2000, 2006; Nguyen *et al.* 2008; Li *et al.* 2010), *Nothoscordum gracile* and *Tulbaghia violacea* were used to root the tree.

### Biogeographical analysis

To perform our analysis, we used the maximum clade credibility (MCC) phylogeny from the ITS data set by BEAST. Distribution areas of *Anguinum* and its close allies (*Caloscordum*, *Vvedenskya*, *Porphyroprason*, and *Melanocrommyum*) were defined according to the World Checklist of Selected Plant Families maintained by the Royal Botanic Gardens, Kew, UK (http://apps.kew.org/wcsp/home.do) and taxonomic and geographical studies of these *Allium* species (e.g. Xu and Kamelin 2000; Dale *et al.* 2002; Choi and Oh 2011). Potential biogeographical scenarios of *Anguinum* were investigated using statistical dispersal–vicariance analysis (S-DIVA; Yu *et al.* 2010) and a maximum likelihood-based method (LAGRANGE; Ree *et al.* 2005; Ree and Smith 2008) implemented in the computer software Reconstruct Ancestral States in Phylogenies (RASP; Yu *et al.* 2015). In the S-DIVA analysis, allowing reconstruction, the number of ancestral areas was restricted to two. The rationale for such a constraint is that vicariance is a proximate consequence of dispersal. Moreover, extant taxa used in the analyses rarely occur in more than two individual areas. The ML inferences of geographic range evolution using LAGRANGE were conducted for the same distribution matrix under the constraints of maximum areas of two. The connectivity between areas was not constrained.

## Results

### Molecular datasets

The ILD test conducted on the combined data matrix of common ITS and chloroplast sequences (*matK *+*  trnH-psbA *+*  rps16*) accessions was signiﬁcant (ILD probability value = 0.01), indicating that the two datasets are heterogeneous (Cunningham 1997). Finally, four different datasets were generated: dataset 1 for *Anguinum* ITS phylogenetic analyses, dataset 2 for *Anguinum* combined chloroplast phylogenetic analyses, dataset 3 for *Allium* divergence time estimation, and dataset 4 for *Anguinum* divergence time estimation. After introducing the necessary gaps, the ITS alignment for dataset 1 was 649 bp in length and resulted in 498 constant characters and 150 variable characters, 110 of which were potentially parsimony-informative; the mean G + C content was 47.5 %. For dataset 2, the combined chloroplast sequences (*matK *+* trnH-psbA *+* rps16*) produced a matrix 2296 bp in length, and for which 2193 characters were constant, 39 autapomorphic and 56 potentially parsimony-informative; the mean G + C content was 31.3 %. The statistics of chloroplast sequences and nuclear ITS are shown in Table 1. For dataset 3, the aligned *rbcL* sequences produced a matrix 1328 bp in length, and for which 741 characters were constant, and 418 potentially parsimony-informative; the mean G + C content was 43.6 %. For dataset 4, the aligned ITS sequences produced a matrix 754 bp in length, and for which 99 characters were constant, and 547 potentially parsimony-informative; the mean G + C content was 46.6 %.

**Table 1.** Characteristics of each gene fragments for dataset 1 and 2.

Information	ITS	*matK*	*trnH*-*psbA*	*rps16*	Combined cpDNA data
Range of length (bp)	642–646	840–855	548–584	813–838	–
Aligned length (bp)	649	855	586	855	2296
Number of variable characters	150	22	25	42	89
Number of parsimony- informative characters	110	17	8	31	56
GC (%)	47.5	30.5	34.6	29.9	31.3
Tree length	162	–	–	–	107
Consistency index (CI)	0.94	–	–	–	0.89
Retention index (RI)	0.98	–	–	–	0.94
Homoplasy index (HI)	0.06	–	–	–	0.11
Substitution model	GTR	GTR	F81+I	F81+I	–

### Phylogeny

#### ITS phylogeny

**Figure 1.**
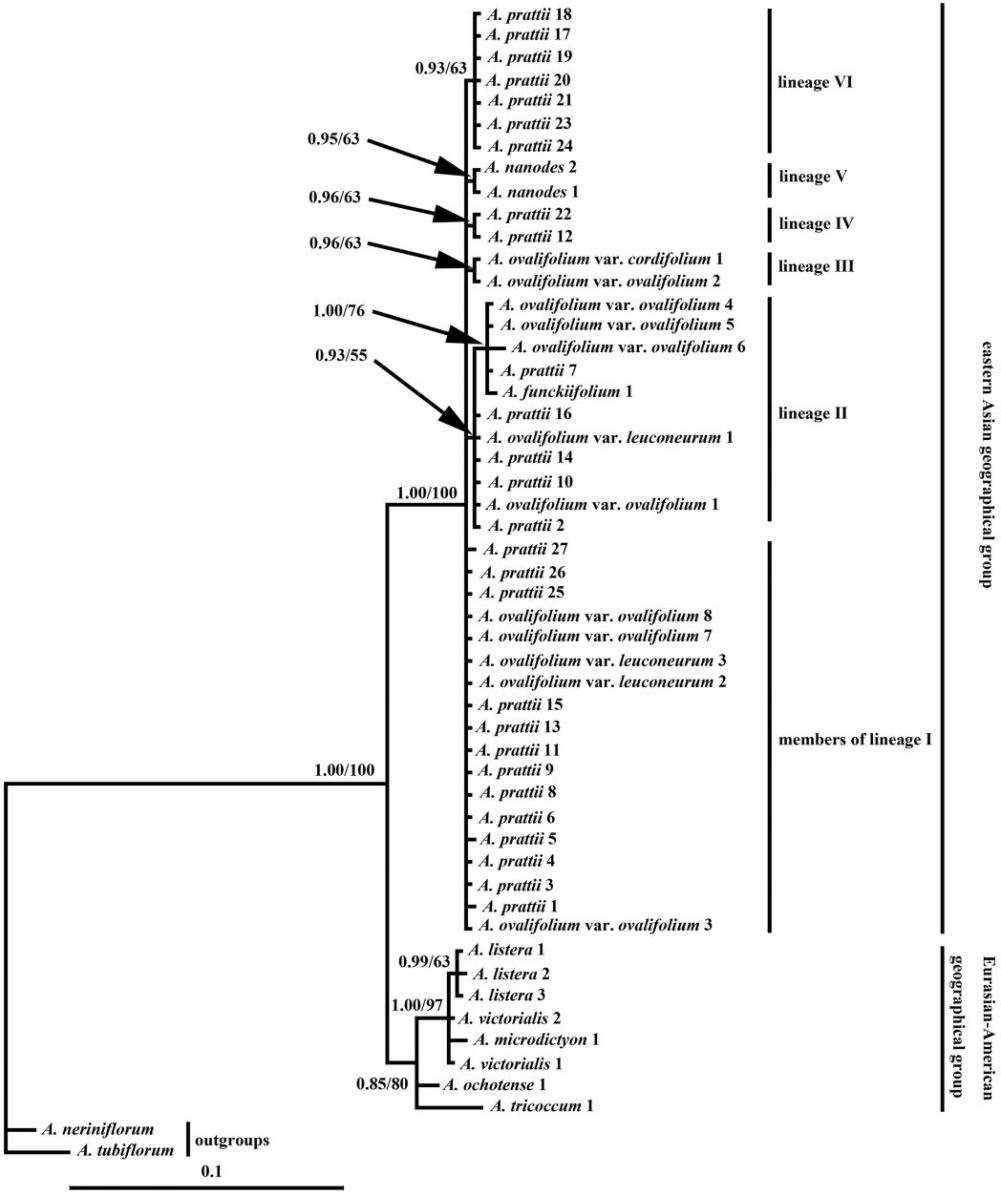
Phylogenetic tree resulting from a Bayesian analysis of the ITS sequences from species of subgenus *Anguinum* plus two outgroups. Branch lengths correspond to the genetic distances (substitutions per site). Values along branches represent Bayesian posterior probabilities (PP) and parsimony bootstrap (BS), respectively. Numbers following taxon names refer to populations identified in the Appendix S1.

For dataset 1, trees inferred from BI and MP showed no significant difference in their topologies, and therefore only the Bayesian tree with posterior probabilities (PP) and bootstrap support values (BS) was shown in Fig. 1. In all analyses, the subgen. *Anguinum* proved to be monophyletic and robustly separated from the outgroup species (PP = 1.00, BS = 100 %). The *Anguinum* contains two sister groups: one with a Eurasian–American distribution (including *A. victorialis*, *A. listera*, *A. microdictyon*, *A. ochotense*, *A. tricoccum* var. *tricoccum*, *A. tricoccum* var. *burdickii*), and the other restricted to eastern Asia (i.e., the Hengduan Mountains and adjacent areas, including *A. ovalifolium* var. *ovalifolium*, *A. ovalifolium* var. *cordifolium*, *A. ovalifolium* var. *leuconeurum*, *A. funckiifolium*, *A. nanodes*, *A. prattii*). Within the Eurasian–American distribution clade, *A. ochotense*, *A. tricoccum*, and three species from Eurasia (*A. listera*, *A. microdictyon*, and *A. victorialis*) form a trichotomy (PP = 0.85, BS = 80 %); three accessions of *A. listera* join together (PP = 0.99, BS = 63 %). Within the eastern Asia distribution clade, species from eastern Asia (*A. ovalifolium* var. *ovalifolium*, *A. ovalifolium* var. *cordifolium*, *A. ovalifolium* var. *leuconeurum*, *A. nanodes*, *A. funckiifolium*, *A. prattii*) form a large basal-most polytomy (PP = 1.00, BS = 100 %), and inside some small polytomies; two accessions of *A. nanodes* grouped together (PP = 0.95, BS = 63 %). In all cases, although there are small clades of alleles from the same taxa, *A. ovalifolium* var. *ovalifolium*, *A. ovalifolium* var. *leuconeurum* and *A. prattii* are non-monophyletic. Considering only one accession involved in the present study, the monophyly/non-monophyly of *A. ovalifolium* var. *cordifolium* and *A. funckiifolium* requires further investigation. Overall, ITS phylogeny is populated by both short internal and terminal branches; therefore, it is not surprising that there are unresolved polytomies.

### Chloroplast sequence phylogeny

**Figure 2.**
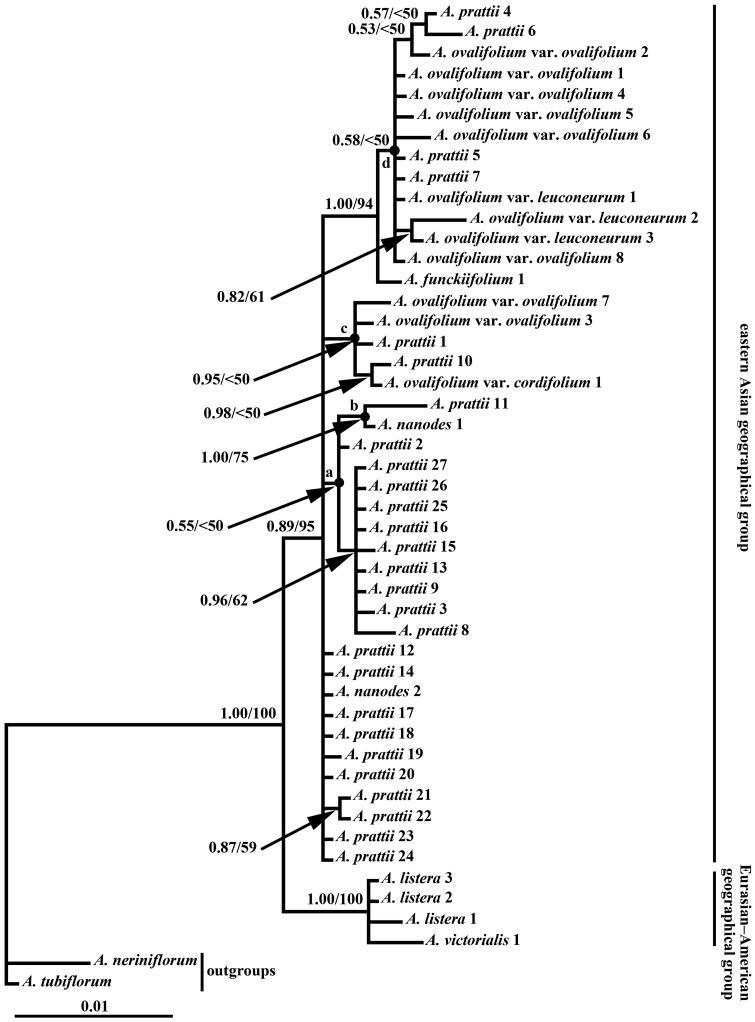
Phylogenetic tree resulting from a Bayesian analysis of the concatenated plastid sequences (*rps16 *+* matK *+* trnH-psbA*) from species of subgenus *Anguinum* plus two outgroups. Branch lengths correspond to the genetic distances (substitutions per site). Values along branches represent Bayesian posterior probabilities (PP) and parsimony bootstrap (BS), respectively. Numbers following taxon names refer to populations identified in the Appendix S1. Letters (a–d) indicate relevant nodes discussed in the text.

In the combined cpDNA analyses, the topology of the Bayesian tree was similar to that of the MP consensus tree. The 50 % majority-rule consensus tree from BI is presented in Fig. 2, with PP and BS support values. The monophyly of subgen. *Anguinum* was also recovered (PP = 1.00, BS = 100 %). Within *Anguinum*, two sister groups are evident. Within the Eurasian–American distribution clade (PP = 1.00, BS = 100 %), accessions of *A. listera* and *A. victorialis* form a polytomy; within the eastern Asia distribution clade (PP = 0.89, BS = 95 %), accessions of *A. ovalifolium* var. *ovalifolium*, *A. ovalifolium* var. *cordifolium*, *A. ovalifolium* var. *leuconeurum*, *A. nanodes*, *A. funckiifolium*, and *A. prattii* form a large basal-most polytomy, and also some small polytomies inside the polytomy. The topological patterns of the combined cpDNA phylogeny are more complex than that of ITS. Accessions from following four taxa did not form monophyletic groups: *A. ovalifolium* var. *ovalifolium*, *A. ovalifolium* var. *leuconeurum*, *A. nanodes*, *A. prattii*, although part of the sequences for the same taxa grouped together. Overall, the cpDNA phylogenetic hypothesis is also rich in polytomies.

### Estimation of divergence time

**Figure 3.**
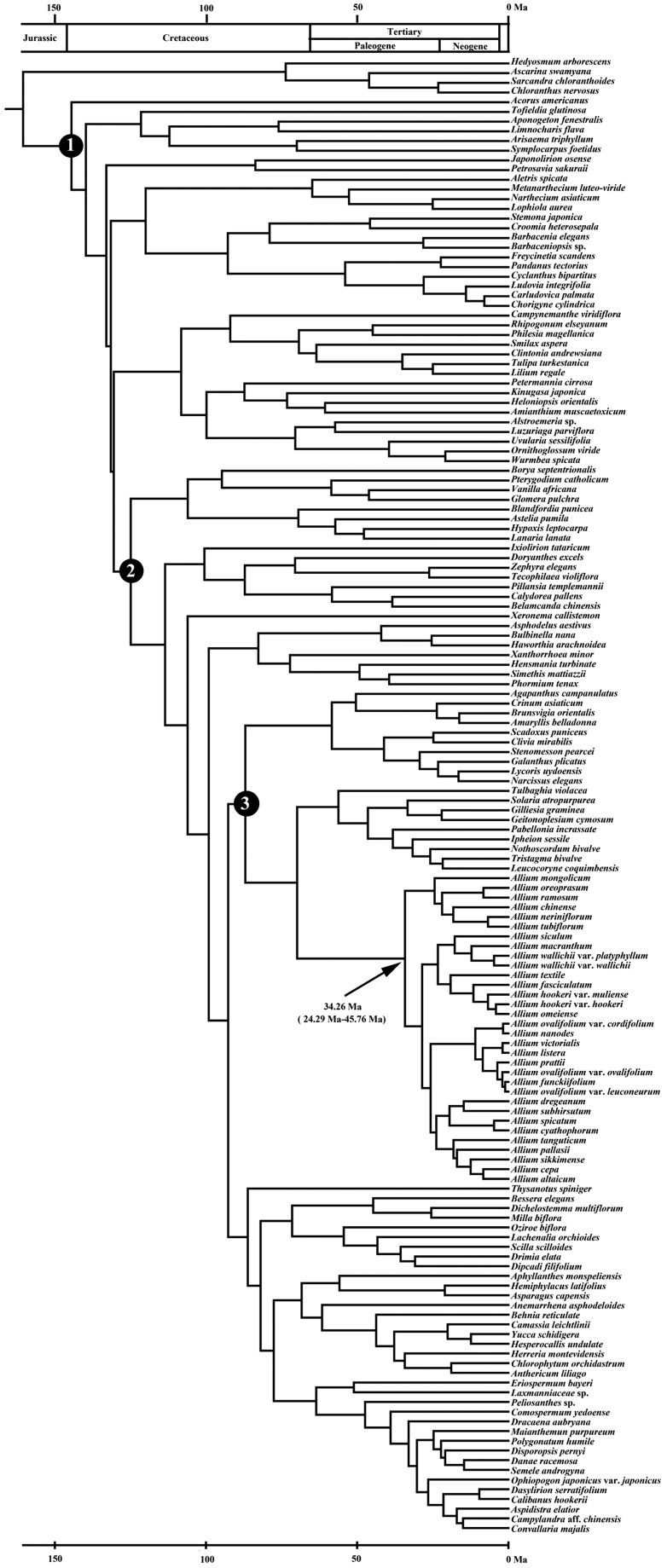
Chronogram of *Allium* from the order Asparagales and other monocots based on *rbcL* data. Divergence times are shown using the computer program BEAST. The tree was rooted using species from order Chloranthales and calibrated using an estimated age of 134 Myr for the age of the crown group of monocots (node 1). The crown group of Asparagales (node 2) and Amaryllidaceae (node 3) was set to be 124.95 Mya and 87 Mya, respectively. The division of the geologic time according to the ‘Geologic Time Scale’ compiled by Walker and Geissman (2009).

**Figure 4.**
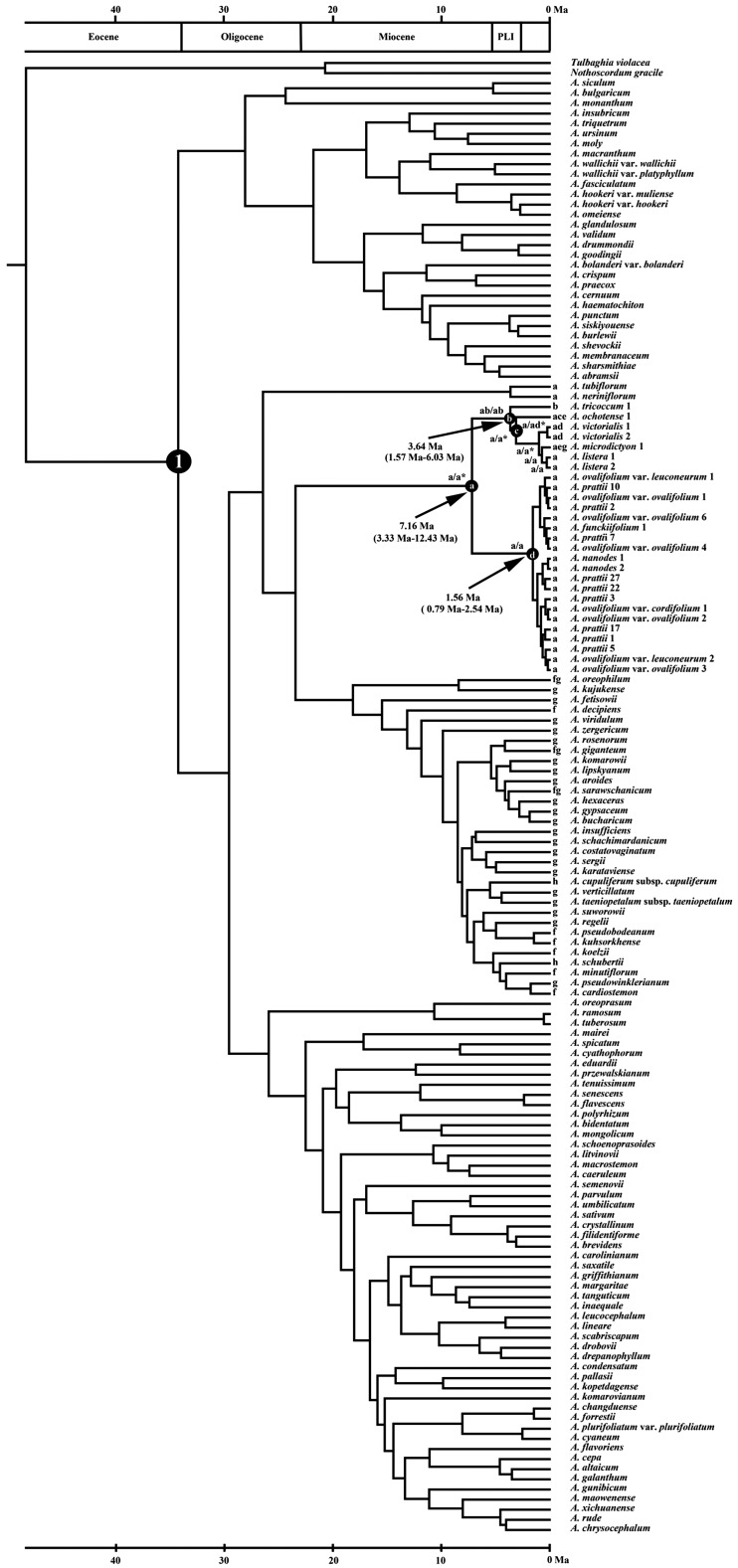
Dated phylogeny for *Anguinum* based on a maximum clade credibility tree obtained from a BEAST analysis of 146 ITS sequences under an uncorrelated lognormal molecular clock. Branch lengths represent millions of years (Ma). Crown age of *Allium* was set to be 34.26 Mya (node 1) based on the estimated date of *Allium* from the order Asparagales and other monocots. The division of the geologic time according to the ‘Geologic Time Scale’compiled by Walker and Geissman (2009). Biogeographic analysis of *Anguinum* was based on the S-DIVA (shown in front of the slashes) and the ML (shown behind the slashes) analyses. The optimal ancestral areas with an asterisk at some node presented under LAGRANGE are the ones with the highest probabilities among the alternatives. Letters (a–d) indicate relevant nodes discussed in the text.

The crown group of genus *Allium* originated during the late Eocene (ca. 34.26 Mya) and then diverged into three clades (Fig. 3). The chronogram and results of divergence-time estimation of *Anguinum* are shown in Fig. 4. The crown group of *Anguinum* originated during the late Miocene (ca. 7.16 Mya). Within *Anguinum*, the divergence of the Eurasian-American alliance clade dates to the Mid-Pliocene (ca. 3.64 Mya), and the origin of the eastern Asian alliance clade to the early Pleistocene (ca. 1.56 Mya).

### Biogeographical analysis

Eight areas were considered for biogeographical analysis of *Anguinum* and its allies: eastern Asia (a), eastern North America (b), western North America (c), Europe and adjacent areas (d), Siberia (e), West Asia and adjacent areas (f), Central Asia (g) and the Mediterranea (h). The inferred historical biogeographic scenarios from analyses using S-DIVA and LAGRANGE are shown in Fig. 4. Both the analyses strongly supported East Asia as the ancestral area of *Anguinum*.

## Discussion

### Sequence divergence and polytomies

For ITS sequences, the average pairwise K2P (Kimura’s two-parameter; Kimura 1980) distance between ingroup *Anguinum* and outgroup *Caloscordum* ranged from 15.73 to 19.58 %. The average pairwise K2P distance within *Anguinum* was very low (range 0.00– 6.37 %), and the average distance was 1.60 %. In the Eurasian-American alliance clade, the average distance was 1.40 % (range 0.00–3.69 %), and in the eastern Asia alliance clade, the average distance was 0.27 % (range 0.00–1.09 %). For chloroplast sequences, the average pairwise K2P distance between ingroup *Anguinum* and outgroup *Caloscordum* ranged from 1.40 to 2.02 %. The average pairwise K2P distance within *Anguinum* was very low (range 0.00–1.17 %), and the average distance was 0.32 %. In the sister group formed by *A. victorialis* and *A. listera*, the average distance was 0.15 % (range 0.00–0.27 %), and in the eastern Asia alliance clade, the average distance was 0.23 % (range 0.00–0.74 %). Overall, ITS data show remarkably low levels of variability within *Anguinum*, and the variation is low in comparison to subgenus *Melanocrommyum* in the same evolutionary line (Gurushidze *et al.* 2008). Chloroplast sequence data also show remarkably low levels of variability within *Anguinum*. Another conspicuous result is the different level of differentiation at the ITS and the chloroplast sequences within and among species of subgen. *Anguinum*. In several species intraspecies diversity is quite high, and several species show almost no interspecies diversity. Low sequence divergence results in an unresolved ITS and chloroplast phylogeny of *Anguinum*, with both short internal and terminal branches; thus, it is not surprising that there are rich in polytomies. Further studies (utilizing low-copy nuclear gene phylogenies and/or employing marker technology that has broad genomic coverage) are needed to obtain more information on species-level relationships of *Anguinum*.

### Phylogenetic implications, incomplete lineage sorting, hybridization/introgression

The results presented here support the earlier finding that subgen. *Anguinum* is monophyletic (Friesen *et al.* 2006; Li *et al.* 2010). In agreement with Friesen *et al.* (2006) and Li *et al.* (2010), two sister groups comprise this subgenus: one with a Eurasian–American distribution and the other restricted to eastern Asia. In the Eurasian–American geographical group, *A. listera*, *A. microdictyon*, and *A. victorialis* are closely related species and form a polytomy, which may indicate that this group diverged rapidly. In the ITS tree, accessions of three *A. listera* populations are clustered together, implying that *A. listera* is monophyletic. In the eastern Asian geographical group, for both the ITS and the cpDNA tree, accessions of *A. ovalifolium* var. *ovalifolium*, *A. ovalifolium* var. *cordifolium*, *A. ovalifolium* var. *leuconeurum*, *A. nanodes*, *A. funckiifolium*, and *A. prattii* form a large basal-most polytomy, and also some small polytomies inside the polytomy. Accessions of two *A. nanodes* populations form a monophyletic group in the ITS tree, and are not clustered together in chloroplast tree. Both ITS tree and chloroplast tree sufficiently indicate that *A. ovalifolium* var. *ovalifolium*, *A. ovalifolium* var. *leuconeurum* and *A. prattii* are non-monophyletic, although there are small clades of alleles from the same taxa. In total, in the eastern Asian geographical group, incongruence between gene trees and morphology-based taxonomies, and incongruence between trees from plastid sequences and nuclear sequences are recovered. This leaves the phylogenetic relationships among the species ambiguous.

Incongruence between molecular data and morphology-based taxonomies (e. g. Gurushidze *et al.* 2008, 2010; Aduse-Poku *et al.* 2009; Waters *et al.* 2010), and incongruence between nuclear and plastid data (e. g. Fehrer *et al.* 2007; Kim and Donoghue 2008; Pelser *et al.* 2010) have been repeatedly reported. Experimental and theoretical studies (e. g. reviewed in Wendel and Doyle 1998; Whitfield and Lockhart 2007; Waterman *et al.* 2009; Gurushidze *et al.* 2010) have shown that some factors can lead to gene trees incongruence and gene tree—species tree incongruence, including stochastic error, systematic error, convergence, evolutionary rate heterogeneity, lineage sorting, and reticulation, namely hybridization, introgression, homoploid and polyploid. When incongruence is recovered among gene trees, the first consideration is whether it is due to inadequate sampling. The second consideration is stochastic error and systematic error. Once these factors are ruled out, biological factors in the evolutionary process may be considered, including convergence and rapid radiation, horizontal gene transfer, hybridization/introgression, and incomplete lineage sorting. Our sampling scheme was designed to cover all taxa of the *Anguinum* eastern Asia geographical group; with the exception of *A. ovalifolium* var. *cordifolium* and *A. funckiifolium*, all remaining taxa were represented by accessions from multiple populations. ITS sequences and three chloroplast sequences (*matK*, *trnH-psbA*, *rps16*) were selected as molecular markers in order to have markers representing different genomic compartments. Phylogenetic analyses of the ITS sequences and the combined chloroplast sequences were performed separately, and in BI, a best-fit model of nucleotide substitution was selected for each chloroplast sequence. Consistency index (CI; Kluge and Farris 1969) and the retention index (RI; Farris 1989) obtained from the parsimony analysis are as follows: CI and RI obtained from the ITS sequences were separately 0.94 and 0.98, and CI and RI obtained from the combined chloroplast sequences were separately 0.89 and 0.94, which illustrate that the levels of homoplasy found in our data set are similar to those of other angiosperm groups (Álvarez and Wendel 2003). Given these tests, it is likely that inadequate sampling as well as stochastic error and systematic error can be ruled out, indicating a biological factor for the observed incongruence.

As a single genetic change in a regulatory region can cause dramatic morphological transformations, which typically are unaccompanied by similar levels of molecular divergence, and the processes that drive molecular divergence can lag behind the phenotypic divergence, the result will be incongruence between molecular phylogenies and morphology (reviewed in Wendel and Doyle 1998). While, data from ITS sequences and three chloroplast sequences provide neither resolved topologies nor congruent hypotheses about species-level relationships in the *Anguinum* eastern Asia geographical group, it is possible that other loci or genomic elements that are more rapidly evolving may provide phylogenetic resolution for this group.

Given the nature of the data, however, it is most probable that incomplete lineage sorting and hybridization/introgression are the main contributing factors to the conflicts found among sequences for the *Anguinum* eastern Asia geographical group. The incongruence occurs between gene trees and morphology-based taxonomies, and between trees from plastid sequences and nuclear ribosomal sequences. These findings are consistent with other studies in *Allium* subgen. *Melanocrommyum* (Gurushidze *et al.* 2010) and other plant genera (e.g., Dobeš *et al.* 2004; Jakob and Blattner 2006; Vargas *et al.* 2009) indicating that the combined effects of incomplete lineage sorting and hybridization/introgression can obscure organismal-level relationships in a phylogenetic framework. Incomplete lineage sorting (ILS) is the persistence of ancestral polymorphisms through speciation events; with time and subsequent extinction of gene lineages, descendant populations will have randomly sorted separate nuclear and organelle sequences (Wendel and Doyle 1998; Avise 2000). ILS is especially likely when species rapidly radiate and population sizes are large (Maddison 1997; Sang 2002; Funk and Omland 2003). A consequence of incomplete lineage sorting is that species will appear to be non-monophyletic. The ITS tree indicates that, *A. nanodes* is monophyletic, while *A. ovalifolium* var. *ovalifolium*, *A. ovalifolium* var. *leuconeurum*, and *A. prattii* are resolved as non-monophyletic. Evidence suggests that nuclear genes move across hybrid boundaries less freely than organellar genes (Rieseberg and Wendel 1993; Soltis and Soltis 1995; Hardig *et al.* 2000), so we conclude that incomplete lineage sorting could most likely account for the difference in the plastid tree vs. the nuclear ITS tree for this group. It cannot be ruled out from these data, however, that ancient reticulation has occurred in the past. During glacial periods in the Quaternary, previously isolated populations of the *Anguinum* eastern Asian geographical group may have come into contact, allowing hybridisation and introgression, resulting in species non-monophyly as evidenced by the DNA data presented here. The combined plastid DNA tree indicated that *A. nanodes*, *A. ovalifolium* var. *ovalifolium*, *A. ovalifolium* var. *leuconeurum*, and *A. prattii* are non-monophyletic, while accessions of partial populations of *A. ovalifolium* var. *ovalifolium*, *A. ovalifolium* var. *cordifolium*, and *A. prattii* are clustered together (Fig. 2, node c), and accessions of partial populations of *A. ovalifolium* var. *ovalifolium*, *A. ovalifolium* var. *leuconeurum*, and *A. prattii* are clustered together (Fig. 2, node d). Above evidence suggests that, the lineage sorting process is either not finished, or that some hybridization is still going on. The coalescence of organelle DNA is four times faster than nuclear genes (Moore 1995), and therefore it is unlikely that the lineage sorting for nuclear genes had been completed if lineage sorting for chloroplast genes is not yet complete. Overall, we propose that the process of lineage sorting is ongoing for both nuclear ribosomal and chloroplast genes in the *Anguinum* eastern Asian geographical group, and any lineage sorting for *A. nanodes* may have been completed, while this process for the other taxa is not yet complete.

Incongruence between phylogenies inferred from nuclear and chloroplast regions, is partially interpreted as being a result of incomplete lineage sorting. However, the incomplete lineage sorting is not the only process that could produce such incongruence. Incongruence between nuclear and chloroplast data, and the sharing of chloroplast haplotypes between species, are also usually interpreted as being a result of hybridization/introgression which results in the chloroplast genome of one species being replaced by that of another species, and such reticulation is easily reflected in the form of cpDNA/nuclear gene tree incongruencies (Anderson 1949; Soltis and Soltis 1995; Rautenberg *et al.* 2010). By means of molecular markers, ‘footprint’ of hybridization/introgression in the *Anguinum* eastern Asian geographical group can be found. A prominent example is phenomenon in *A. nanodes*. Lineage sorting for ITS in this species has been completed (Fig.1), but in chloroplast tree (Fig. 2), *A. nanodes* 1 and *A. nanodes* 2 are not clustered together. *A. nanodes* 1 is clustered together with multiple populations of *A. prattii* (Fig. 2, node a) and is the sister group with *A. prattii* 11 from close localities (Fig. 2, node b), which fully demonstrate that after complete lineage sorting, subsequent hybridization or introgression occurred, and the choloplast genome of *A. nanodes* 1 was replaced by that of *A. prattii* from close localities. It is thus possible that currently delimited morphospecies had insufficient time to develop reproductive barriers, thus promoting hybridisation/introgression. Previous studies (Jing *et al.* 1999; Xu and Kamelin 2000) suggested that polyploids existed in *Allium ovalifolium* var. *ovalifolium* (2n = 16, 24) and *A. prattii* (2n = 16, 32), which perhaps also imply hybridization/introgression exists in the *Anguinum* eastern Asian geographical group. While it may be possible to detect hybridization/introgression by means of multiple base calls in ITS sequence electropherograms (Noyes 2006; Carine *et al.* 2007), the homogenous nature of the ITS data we obtain means that detecting hybridization/introgression using this approach is not possible. Young species groups in particular should be affected by incomplete lineage sorting while its influence should decrease with increasing time due to the gradual loss of polymorphisms and fixation of lineage-specific alleles (Maddison 1997; Wendel and Doyle 1998). As hybridization/introgression also occurs mostly between young and reproductively not completely isolated species (Comes and Abbott 2001; Ramdhani *et al.* 2009), incomplete lineage sorting and hybridization/introgression may not be mutually exclusive. It is much more difficult to distinguish between incomplete lineage sorting and hybridization/introgression because these processes can produce almost identical outputs at the level of tree discordances (Wendel and Doyle 1998; Ramdhani *et al.* 2009; Lu *et al.* 2010). With the existing information it is impossible to distinguish the relative impact of incomplete lineage sorting vs. hybridization/introgression in the *Anguinum* eastern Asian geographical group.

### A hypothesis of the evolutionary history of *anguinum*

Our analyses revealed that the crown group of *Anguinum* originated during the late Miocene (ca. 7.16 Mya), and eastern Asia was the ancestral area for *Anguinum* (node a), where it underwent duplication, and gave rise to two different lineages. during the Mid-Pliocene (ca. 3.64 Mya), one of them, the ancestor of the Eurasian-American alliance clade began to diverge (node b), one descendant probably dispersed to the northeastern North America by the BLB or by the long distance dispersal and diverged into two varieties (*A. tricoccum* var. *tricoccum* and *A. tricoccum* var. *burdickii*); the other descendant stayed in eastern Asia (node c) and began to diverge at ca. 3.09 Mya, in which *A. ochotense* is the earliest-branching species and gradually dispersed to western North America (Attu Island) and Siberia—while ancestor of the remaining species (*A. listera*, *A. microdictyon*, and *A. victorialis*) originated at the Mid-Pleistocene (ca. 0.98 Mya), in which *A. victorialis* is the early-branching species and gradually dispersed to Europe and *A. microdictyon* gradually dispersed to Siberia and Central Asia. Compared to the origin of the Eurasian-American alliance clade, the origin of the eastern Asian alliance (eastern Himalaya and areas south of the Qinlin Mountains of China) clade (node d) is a relatively recent event, which began to diverge at the early Pleistocene (ca. 1.56 Mya), and *A. nanodes* originated at the late Pleistocene (ca. 0.14 Mya). Geological estimates date the start of the uplift of the Himalayas and Hengduan Mountains unequivocally within the Late Tertiary/mid-Miocene (ca. 15–10 Ma; Royden *et al.* 2008), and episodes of uplift probably continued throughout the Late Pliocene (ca. 3 Ma) and well into the Quaternary (Li *et al.* 1979; Zhou *et al.* 2006). Severe climatic oscillations had dramatic effects on the evolution and distribution of plants in this region (Sun 2002; Qiu *et al.* 2011). It is inferred that during the late Pliocene/Early Pleistocene, with cooling climates and the uplift of the Himalayas and Hengduan Mountains, the ancestor of the *Anguinum* eastern Asian lineage underwent a very recent radiation, and gave rise to several closely related species constituting *A. ovalifolium* var. *ovalifolium*, *A. ovalifolium* var. *cordifolium*, *A. ovalifolium* var. *leuconeurum*, *A. funckiifolium*, *A. nanodes*, and *A. prattii*. Recent rapid radiations could result in morphospecies appearing non-monophyletic in DNA-based studies (reviewed by Seehausen 2004; Ramdhani *et al.* 2009, Lu *et al.* 2010), as we have found in the *Anguinum* eastern Asian lineage. These taxa may have had insufficient time to develop reproductive barriers following recent radiation, thus promoting hybridization (Ramdhani *et al.* 2009), and also insufficient time to finish the lineage sorting process. Due to rapid radiation, phylogenetically inferred internodes on gene trees may be short and difficult to resolve with confidence. This short interior branch phenomenon may be also a common cause of phylogenetic incongruence (Wendel and Doyle 1998). Hybridization appears to facilitate the ability of some plant species to adapt to or evolve in response to changes in climate (Bradshaw and McNeilly 1991). It is inferred that, hybridization/introgression in the *Anguinum* eastern Asian lineage could be promoted by the ecological heterogeneity and rapidly changing environment resulting from the intense uplift of the Himalaya, resulting in rampant species non-monophyly, and also partially explaining the inconguence between ITS tree and cpDNA tree.

## Sources of Funding

This work was supported by the National Natural Science Foundation of China (Grant Nos. 31270241, 31470009, 31570198, 31460051), and the National Specimen Information Infrastructure, Educational Specimen Sub-Platform (Web, http://mnh.scu.edu.cn/).

## Contributions by the Authors

Qin-Qin Li conceived the idea and performed the experiment, and then wrote the manuscript. Xian-Qin Wei conducted 1 years of ﬁeldwork. De-Qing Huang made valuable comments on the manuscript. Xing-Jin He and Song-Dong Zhou made valuable comments on the manuscript and provided technical support in the experiments.

## Conflicts of Interest Statement

None declared.

## Supplementary Material

Supplementary Data

## Supporting Information

The following additional information is available in the online version of this article —

 **Appendix S1.** *Anguinum* material of 50 samples plus two species from *Caloscordum* (*A. neriniflorum*, *A. tubiflorum*), with corresponding locality, voucher information, GenBank reference numbers.

 **Appendix S2.** Taxa, and GenBank accession numbers for all *rbcL* sequences used in the present study.

 **Appendix S3.** Taxa, references, and GenBank accession numbers for all ITS sequences used in the *Anguinum* divergence time estimation.
