# Supplementary material for: Molecular phylogeny, divergence time estimates and historical biogeography within one of the world's largest monocot genera
Source: AoB Plants. 2016 Aug 8;8:plw041. doi: 10.1093/aobpla/plw041 (PMC4976397; doi:10.1093/aobpla/plw041)
Supplement: Supplementary Data [file supp_8_plw041_index.html]

Molecular phylogeny, divergence time estimates and historical biogeography within one of the world's largest monocot genera — Supplementary Data 

# Molecular phylogeny, divergence time estimates and historical biogeography within one of the world's largest monocot genera

## Supplementary Data

files

- Supplementary Data - zip file
