# Supplementary material for: Molecular phylogeny, divergence time estimates and historical biogeography within one of the world's largest monocot genera
Source: AoB Plants. 2016 Aug 8;8:plw041. doi: 10.1093/aobpla/plw041 (PMC4976397; doi:10.1093/aobpla/plw041)
Supplement: Supplementary Data [file supp_plw041_suppl_data.zip › aobplants-15020-T-s02.doc]

# OPEN ACCESS – RESEARCH ARTICLE

# Molecular phylogeny, divergence time estimates, and historical biogeography within one of the world's largest monocot genera

**Qin-Qin Li ^1, 2^, Song-Dong Zhou ^1^, De-Qing Huang^1^, Xing-Jin He ^1,^ *, Xian-Qin Wei ^1^**

^1^ School of Life Sciences, Sichuan University, Chengdu 610064, Sichuan, China

^2^ College of Life Science and Technology, Inner Mongolia Normal University, Hohhot, Inner Mongolia 010022, China

*Corresponding author

Corresponding author’s e-mail address: xjhe@scu.edu.cn

Running head: Phylogeny and biogeography of *Allium* subgenus *Anguinum*.
