# Supplementary material for: Molecular phylogeny, divergence time estimates and historical biogeography within one of the world's largest monocot genera
Source: AoB Plants. 2016 Aug 8;8:plw041. doi: 10.1093/aobpla/plw041 (PMC4976397; doi:10.1093/aobpla/plw041)
Supplement: Supplementary Data [file supp_plw041_suppl_data.zip › aobplants-15020-T-s01.doc]

Supporting Information

Appendix S1. *Anguinum* material of 50 samples plus two speices from *Caloscordum* (*A. neriniflorum*, *A. tubiflorum*), with corresponding locality, voucher information, GenBank reference numbers for eastern Aisa *Anguinum*. For the voucher specimens of our tested material, except small part (collection voucher in bold) were deposited in the herbarium of Institute of Botany, Chinese Academy of Sciences (PE), all the others were deposited in the herbarium of the Sichuan University (SZ). Population numbers are given after species names. A dash indicates the region was not included in the present study. GenBank accession numbers in bold indicate those downloaded from the GenBank. All the other sequences are our own accessions.

| *Taxon* | locality | Collection voucher | ITS | *matK* | *trnH-psbA* | *rps16* |
| --- | --- | --- | --- | --- | --- | --- |
| *A. funckiifolium* Hand.-Mazz. 1 | Wanrenkeng, Wushan, Chongqing, China | L Q-Q 2010071601 | HQ690557 | HQ690692 | HQ690607 | KP207739 |
| *A. listera* Stearn 1 | Dongfeng, Jilin, China | Tuliguer 91001 | GQ181063 | HQ690682 | HQ690597 | GU566668 |
| *A. listera* Stearn 2 | Xiaowutaishan, Yuxian, Hebei, China | Zhao L-H  10071904 | HQ690558 | HQ690694 | HQ690609 | KP207741 |
| *A. listera* Stearn 3 | Huashan, Shanxi, China | Zhao L-H &  LQ-Q10081501 | HQ690559 | HQ690704 | HQ690619 | KP207751 |
| *A. microdictyon* Prokh. 1 | B. G. Kirovsk, Altai, Russia | Tax 1300 | **AJ411859** | – | – | – |
| *A. nanodes* Airy-Shaw 1 | Bowashan, Daocheng, Sichuan , China | Li Q-Q BW09080101 | GU566615 | HQ690687 | HQ690602 | GU566666 |
| *A. nanodes* Airy-Shaw 2 | Wumingshan, Xiangcheng, Sichuan, China | Li Q-Q 092302 | HQ690554 | HQ690688 | HQ690603 | KP207735 |
| *A. neriniflorum* (Herb.) G.Don | Songshan, Beijing, China | W Z-X 2010071506 | HQ690560 | HQ690691 | HQ690606 | KP207738 |
| *A. ochotense* Prokh. 1 | unknown | KNA-E20 (KH) | **GQ412223** | – | – | – |
| *A. ovalifolium* var. *cordifolium* (J.M.Xu) J.M.Xu 1 | Laokangmao, Shua jing si, Hong yuan, Sichuan, China | Li Q-Q & Wei X-Q 2009061002 | HQ690555 | HQ690689 | HQ690604 | KP207736 |
| *A. ovalifolium* var. *leuconeurum* (J.M.Xu) J.M.Xu 1 | Dabanzhao, Lixian, Sichuan, China | Li Q-Q 09060901 | GU566616 | HQ690684 | HQ690599 | GU566665 |
| *A. ovalifolium* var. *leuconeurum* (J.M.Xu) J.M.Xu 2 | Shanjiaoba, Lixian, Sichuan, China | Li Q-Q & Wei X-Q 2009060905 | KP207687 | HQ690685 | HQ690600 | KP207733 |
| *A. ovalifolium* var. *leuconeurum*  (J.M.Xu) J.M.Xu 3 | Dacaoba, Fubianxiang, Xiaojin, Sichuan, China | Li Q-Q & Wei X-Q 2009061305 | HQ690553 | HQ690686 | HQ690601 | KP207734 |
| *A. ovalifolium* Hand.-Mazz. var. *ovalifolium* 1 | Dabanzhao, Lixian, Sichuan, China | Li Q-Q & Wei X-Q 2009060802 | HQ690520 | HQ690660 | HQ690575 | KP207713 |
| *A. ovalifolium* Hand.-Mazz. var. *ovalifolium* 2 | Donggouxipo, Shua jing si, Hong yuan, Sichuan, China | Li Q-Q & Wei X-Q 2009061003 | HQ690521 | HQ690661 | HQ690576 | KP207714 |
| *A. ovalifolium* Hand.-Mazz. var. *ovalifolium* 3 | Huishuigou, Fubianxiang, Xiaojin, Sichuan, China | Li Q-Q & Wei X-Q 2009061301 | HQ690522 | HQ690662 | HQ690577 | KP207715 |
| *A. ovalifolium* Hand.-Mazz. var. *ovalifolium* 4 | Xinglongshan, Yuzhong, Gansu, China | Wei X-Q 2009072507 | HQ690526 | HQ690666 | HQ690581 | KP207719 |
| *A. ovalifolium* Hand.-Mazz. var. *ovalifolium* 5 | Shangbaiyun, Taibaishan, Shanxi, China | Li Q-Q 09080805 | HQ690527 | HQ690667 | HQ690582 | KP207720 |
| *A. ovalifolium* Hand.-Mazz. var. *ovalifolium* 6 | Huanglong, Songpan, Sichuan, China | Ma X-G C09091901 | HQ690528 | HQ690668 | HQ690583 | KP207721 |
| *A. ovalifolium* Hand.-Mazz. var. *ovalifolium* 7 | Huishuigou, Fubianxiang, Xiaojin, Sichuan, China | Li Q-Q & Wei X-Q 2009061302 | HQ690556 | HQ690690 | HQ690605 | KP207737 |
| *A. ovalifolium* Hand.-Mazz. var. *ovalifolium* 8 | Guanwushan, Jiangyou, Sichuan, China | Ma X-G C1051401 | HQ690529 | HQ690693 | HQ690608 | KP207740 |
| *A. prattii* C.H.Wright ex Hemsley 1 | Sandaoqiao, Kangding, Sichuan, China | Li Q-Q & Wei X-Q2009062701 | HQ690523 | HQ690663 | HQ690578 | KP207716 |
| *A. prattii* C.H.Wright ex Hemsley 2 | Lushan, Xichang, Sichuan, China | Gao Y-D G20090726-5 | HQ690524 | HQ690664 | HQ690579 | KP207717 |
| *A. prattii* C.H.Wright ex Hemsley 3 | Shuduhu, Xianggelila, Yunan, China | Li Q-Q BT09072803 | HQ690525 | HQ690665 | HQ690580 | KP207718 |
| *A. prattii* C.H.Wright ex Hemsley 4 | Shanjiaoba, Lixian, Sichuan, China | Li Q-Q & Wei X-Q 2009060903 | HQ690531 | HQ690669 | HQ690584 | KP207722 |
| *A. prattii* C.H.Wright ex Hemsley 5 | Balangshan, Xiaojin, Sichuan, China | Li Q-Q 2008081603 | GQ181087 | HQ690670 | HQ690585 | GU566667 |
| *A. prattii* C.H.Wright ex Hemsley 6 | Kuailegou, Fengtongzhai, Baoxing, Sichuan, China | Li Q-Q & Wei X-Q 2009062101 | HQ690532 | HQ690671 | HQ690586 | KP207723 |
| *A. prattii* C.H.Wright ex Hemsley 7 | Wengongmiao, Taibaishan, Shanxi, China | Li Q-Q 09080903 | HQ690533 | HQ690672 | HQ690587 | KP207724 |
| *A. prattii* C.H.Wright ex Hemsley 8 | Bitahai, Xianggelila, Yunan, China | Li Q-Q BT09072810 | HQ690534 | HQ690673 | HQ690588 | KP207725 |
| *A. prattii* C.H.Wright ex Hemsley 9 | Houshan, Yicun, Xianggelila, Yunan , China | Li Q-Q YC09072921 | HQ690535 | HQ690674 | HQ690589 | KP207726 |
| *A. prattii* C.H.Wright ex Hemsley 10 | Bowashan, Daocheng, Sichuan, China | Li Q-Q BW09080102 | HQ690537 | HQ690675 | HQ690590 | KP207727 |
| *A. prattii* C.H.Wright ex Hemsley 11 | Pamuling, Yajiang, Sichuan, China | Xu Y P090809 | HQ690540 | HQ690676 | HQ690591 | KP207728 |
| *A. prattii* C.H.Wright ex Hemsley 12 | Zuogong, Tibet, China | Zhang Y-C B06 | HQ690536 | HQ690677 | HQ690592 | KP207729 |
| *A. prattii* C.H.Wright ex Hemsley 13 | Bitahai, Xianggelila, Yunan, China | Li Q-Q BT09072804 | HQ690530 | HQ690678 | HQ690593 | KP207730 |
| *A. prattii* C.H.Wright ex Hemsley 14 | Bowashan, Daocheng, Sichuan, China | Li Q-Q BW09080104 | HQ690538 | HQ690679 | HQ690594 | KP207731 |
| *A. prattii* C.H.Wright ex Hemsley 15 | Bitahai, Xianggelila, Yunan, China | Li Q-Q BT09072812 | HQ690541 | HQ690681 | HQ690596 | KP207732 |
| *A. prattii* C.H.Wright ex Hemsley 16 | Xiayadong, Yadong, Tibet, China | Yu Y 1611 | HQ690539 | HQ690695 | HQ690610 | KP207742 |
| *A. prattii* C.H.Wright ex Hemsley 17 | Yakou, Changdu to Leiwuqi, Tibet, China | **Gao Y-D 10-1237** | HQ690544 | HQ690696 | HQ690611 | KP207743 |
| *A. prattii* C.H.Wright ex Hemsley 18 | 214 guodao 850 KM, Yushu, Qinghai, China | **Gao Y-D 10-1037** | HQ690545 | HQ690697 | HQ690612 | KP207744 |
| *A. prattii* C.H.Wright ex Hemsley 19 | Leijilashan, Jiangda, Tibet, China | Gao Y-D G2010081004 | HQ690546 | HQ690698 | HQ690613 | KP207745 |
| *A. prattii* C.H.Wright ex Hemsley 20 | Anbalashan, Ziduo, Qinghai, China | **Gao Y-D 10-947** | HQ690547 | HQ690699 | HQ690614 | KP207746 |
| *A. prattii* C.H.Wright ex Hemsley 21 | Lalongcuohaizi, Baiyu to Ganzi, Sichuan, China | **Gao Y-D 20100727** | HQ690548 | HQ690700 | HQ690615 | KP207747 |
| *A. prattii* C.H.Wright ex Hemsley 22 | Awangxiang to Dongshaxiang, Gongjue, Tibet, China | **Gao Y-D 10-1918** | HQ690542 | HQ690701 | HQ690616 | KP207748 |
| *A. prattii* C.H.Wright ex Hemsley 23 | Changdu to Tuoba 317 xian 1215 KM, Changdu, Tibet, China | **Gao Y-D 20100809** | HQ690549 | HQ690702 | HQ690617 | KP207749 |
| *A. prattii* C.H.Wright ex Hemsley 24 | Zichala, Leiwuqi, Tibet, China | **Gao Y-D 10-1434** | HQ690543 | HQ690703 | HQ690618 | KP207750 |
| *A. prattii* C.H.Wright ex Hemsley 25 | Mahuangba, Lijiang, Yunnan, China | Li Q-Q MH09072524 | HQ690550 | HQ690705 | HQ690620 | KP207752 |
| *A. prattii* C.H.Wright ex Hemsley 26 | Wenbishan, Lijiang, Yunnan, China | Liu S & Gao P 10903 | HQ690551 | HQ690706 | HQ690621 | KP207753 |
| *A. prattii* C.H.Wright ex Hemsley 27 | Daxueshanyakou, Xianggelila, Yunnan, China | Gao Y-D 20100718 | HQ690552 | HQ690707 | HQ690622 | KP207754 |
| *A. tricoccum* Sol. 1 | Glencoe, J. F. Swenson, USA | Tax 2582 | **AJ411917** | – | – | – |
| *A. tubiflorum* Rendle | Huashan, Shanxi, China | Liao C-Y C072804 | GU566622 | HQ690680 | HQ690595 |  |
| *A. victorialis* L. 1 | Dunhua, Jilin, China | Tuliguer 91002 | GQ181082 | HQ690683 | HQ690598 | GU566669 |
| *A. victorialis* L. 2 | Caucasus, Georgia | Tax 2673 | **AJ411919** | – | – | – |

Appendix S2. Taxa, and GenBank accession numbers for all *rbcL* sequences used in the present study.Taxa in bold are our own accessions.

*Acorus americanus* DQ069499; *Agapanthus campanulatus* Z69220; *Aletris spicata* AB088834; *Allium altaicum* Z69204; *Allium cepa* EF613555; ***Allium chinense*** —KP207701; Chengdu, Sichuan, China; Li Q-Q 09011521; ***Allium cyathophorum***—KP207698; Zhongdian, Yunnan, China; Xu J-M 93-17; *Allium dregeanum* AM234798; ***Allium fasciculatum***—KP207689; Dazi, Xizang, China; Tu Y-L *et al.* 94-9; ***Allium funckiifolium***—KP207694; Wanrenkeng, Wushan, Chongqing; L Q-Q 2010071601; ***Allium hookeri* var. *hookeri***—KP207690; Lijiang, Yunnan, China; Li Q-Q 09072702; ***Allium hookeri* var. *muliense***—KP207695; Zhongdian ,Yunnan, China; Xu J-M 93-25; ***Allium listera***—KP207693; Dongfeng, Jilin, China; Tuliguer 91001; ***Allium macranthum***—KP207702; Zhongdian, Yunnan, China; Xu J-M 93-23; ***Allium mongolicum***—KP207699; Saihan, Nei Mongol, China; He X-J *et al.* 97802; ***Allium nanodes*** —KP207696; Daocheng, Sichuan, China; Li Q-Q 09080101; ***Allium neriniflorum***— KP207688; Songshan, Beijing, China; W Z-X 2010071506; ***Allium omeiense***— KP207704; Emei Mountain, Sichuan, China; Xu J-M 91-01; ***Allium oreoprasum***—KP207705; Tuoli, Xinjiang, China; He X-J & Zhang X-L 97619; ***Allium ovalifolium* var. *cordifolium***—KP207700; Laokangmao, Shuajingsi, Hong yuan, Sichuan, China; Li Q-Q & Wei X-Q 2009061002; ***Allium ovalifolium var. leuconeurum***— KP207691; Shanjiaoba, Lixian, Sichuan, China; Li Q-Q & Wei X-Q 2009060905; ***Allium ovalifolium var. ovalifolium***—KP207706; Dabanzhao, Lixian, Sichuan, China; Li Q-Q & Wei X-Q 2009060802; ***Allium pallasii***—KP207697; Emin, Xinjiang, China; He X-J & Zhang X-L 97603; ***Allium prattii***—KP207709; Shanjiaoba, Lixian, Sichuan, China; Li Q-Q & Wei X-Q 2009060903; ***Allium ramosum***—KP207710; Daqing Mountain, Nei Mongol, China; He X-J *et al.* 97812; *Allium siculum* Z69200; ***Allium sikkimense***—KP207711; Daocheng, Sichuan, China; Li Q-Q 09080105; ***Allium spicatum***—KR902509; Rikaze, Xizang, China; Huang D-Q XZ2011082609; ***Allium tanguticum***—KP207712; Qinghaihu, Qinghai, China; Ma X-G 09080301; *Allium textile* AY149372; ***Allium tubiflorum***—KP207708; Huashan, Shanxi, China; Liao C-Y C072804; ***Allium victorialis***—KP207703; Dunhua, Jilin, China; Tuliguer 91002; ***Allium wallichii* var. *platyphyllum***—KP207692; Lijiang, Yunnan, China; Li Q-Q 09072503; ***Allium wallichii* var. *wallichii***—KP207707; Kangding, Sichuan, China; Li Q-Q 2008081203; *Allium subhirsutum* Z69205; *Alstroemeria* sp. Z77254; *Amaryllis belladonna* Z69219; *Amianthium muscaetoxicum* AJ417895; *Anemarrhena asphodeloides* Z77251U; *Anthericum liliago* Z69225; *Aphyllanthes monspeliensis* Z77259; *Aponogeton fenestralis* AB088808; *Arisaema triphyllum* AY298817; *Ascarina swamyana* AY236843; *Asparagus capensis* AM234843; *Asphodelus aestivus* AJ512314; *Aspidistra elatior* Z77269U; *Astelia pumila* AF307906; *Barbacenia elegans* AJ131946; *Barbaceniopsis* sp. AY298819; *Behnia reticulate* Z69226; *Belamcanda chinensis* AJ307078; *Bessera elegans* Z69215; *Blandfordia punicea* Z73694; *Borya septentrionalis* Z77262; *Brunsvigia orientalis* AM234805; *Bulbinella nana* AJ512325; *Calibanus hookerii* Z77276; *Calydorea pallens* AJ309682; *Camassia leichtlinii* Z69238; *Campylandra* aff. *chinensis* AB089638; *Campynemanthe viridiflora* AJ276349; *Carludovica palmata* AF197596; *Chloranthus nervosus* AY236841; *Chlorophytum orchidastrum* Z77257; *Chorigyne cylindrica* AY298823; *Clintonia andrewsiana* AB056850; *Clivia mirabilis* AM234806; *Comospermum yedoense* Z73679; *Convallaria majalis* D28334; *Crinum asiaticum* HM640488; *Croomia heterosepala* D28154; *Cyclanthus bipartitus* AY007660; *Danae racemosa* Z73708; *Dasylirion serratifolium* AB029847; *Dichelostemma multiflorum* Z69211; *Dipcadi filifolium* AM902303; *Disporopsis pernyi* D17373; *Doryanthes excels* Z73697; *Dracaena aubryana* Z77270; *Drimia elata* AM234987; *Eriospermum bayeri* Z77277; *Freycinetia scandens* AF206770; *Galanthus plicatus* Z69218; *Geitonoplesium cymosum* AF116997; *Gilliesia graminea* Z69208; *Glomera pulchra* AF518053; *Haworthia arachnoidea* AM234844; *Hedyosmum arborescens* L12649; *Heloniopsis orientalis* AJ417894; *Hemiphylacus latifolius* Z73688; *Hensmania turbinate* FJ707501; *Herreria montevidensis* Z69230; *Hesperocallis undulate* AY561251; *Hypoxis leptocarpa* Z73702; *Ipheion sessile* Z69201; *Ixiolirion tataricum* Z73704; *Japonolirion osense* AB088835; *Kinugasa japonica* D28157; *Lachenalia orchioides* AM234989; *Lanaria lanata* Z77313; *Laxmanniaceae* sp. GQ423729; *Leucocoryne coquimbensis* Z69199; *Lilium regale* AB088816; *Limnocharis flava* AB088807; *Lophiola aurea* AB088836; *Ludovia integrifolia* L10251; *Luzuriaga parviflora* AF307925; *Lycoris uydoensis* HM640486; *Maianthemun purpureum* EU850081; *Metanarthecium luteo-viride* AB088837; *Milla biflora* Z69216; *Narcissus elegans* AF116972; *Narthecium asiaticum* AB088838; *Nothoscordum bivalve* Z69202; *Ophiopogon japonicus* var. *japonicus* AB029841; *Ornithoglossum viride* AM234963; *Oziroe biflora* Z69222; *Pabellonia incrassate* Z69209; *Pandanus tectorius* M91632; *Peliosanthes* sp. Z77273; *Petermannia cirrosa* AY465714; *Petrosavia sakuraii* AB088839; *Philesia magellanica* Z77302; *Phormium tenax* Z69232; *Pillansia templemannii* AJ309671; *Polygonatum humile* AB009947; *Pterygodium catholicum* AY368346; *Rhipogonum elseyanum* Z77309; *Sarcandra chloranthoides* AY236833; *Scadoxus puniceus* AM234808; *Scilla scilloides* D28161; *Semele androgyna* Z77275; *Simethis mattiazzii* Z69231; *Smilax aspera* GU945049; *Solaria atropurpurea* Z69207; *Stemona japonica* AJ131948; *Stenomesson pearcei* Z69217; *Symplocarpus foetidus* L10247; *Tecophilaea violiflora* Y17337; *Tofieldia glutinosa* DQ182340; *Thysanotus spiniger* Z69236; *Tristagma bivalve* Z69206; *Tulbaghia violacea* Z69203; *Tulipa turkestanica* AB037378; *Uvularia sessilifolia* AB009948; *Vanilla Africana* AF074239; *Wurmbea spicata* AM234964; *Xeronema callistemon* Z69235; *Xanthorrhoea minor* Y17339; *Yucca schidigera* DQ069504; *Zephyra elegans* Y17340.

Appendix S3. Taxa, references, and GenBank accession numbers for all ITS sequences used in the *Anguinum* divergence time estimation. Sequences in bold are our own accessions. ^1^Dubouzet and Shinoda (1998); ^2^Dubouzet and Shinoda (1999); ^3^Friesen *et al.* (2000); ^4^Friesen *et al.* (2006); ^5^Gurushidze *et al.* (2007); ^6^Gurushidze *et al.* (2008); ^7^Nguyen *et al.* (2008); ^8^Jang *et al.* (unpublished).

*Allium*: *A. abramsii* (Ownbey & Aase) McNeal EU096131^7^; *A. altaicum* Pall. —**GQ181094**; Burqin, Xinjiang, China; He X-J & Zhang X-L 97643; *A. aroides* Popov & Vved. FM177259^6^; *A. bidentatum* Fisch. ex Prokh. & Ikonn.-Gal. —**GQ181096**; Ximeng, Nei Mongol, China; He X-J *et al*. 97813; *A. bolanderi* S.Watson var. *bolanderi* EU096139^7^; *A. brevidens* Vved. AJ412721^4^; *A. bucharicum* Regel FM177275^6^; *A. bulgaricum* (Janka) Prodan AJ412747^4^; *A. burlewii* Davidson EU096142^7^; *A. caeruleum* Pall. —**GQ181064**; Yumin, Xinjiang, China; He X-J & Zhang X-L 97609; *A. cardiostemon* Fisch. & C.A.Mey. FM177277^6^; *A. carolinianum* DC. —**GQ181097**; Wμlumuqi, Xinjiang, China; He X-J & Zhang X-L 97656; *A. cepa* L. AM418370^5^; *A. cernuum* Roth AF037622^1^; *A. changduense* J.M.Xu—**HQ690571**; Leiwuqi, Tibet, China; Gao Y-D 10-1263; *A. chrysocephalum* Regel—**HQ690287**; Lianhuashan, Gansu, China; Wei X-Q 2009072604; *A. condensatum* Turcz. —**HQ690573**; Jiamusi, Heilongjiang, China; Wang C-B 0922; *A. costatovaginatum* Kamelin & Levichev FM177286^6^; *A. crispum* Greene EU096147^7^; *A. crystallinum* Vved. AJ412724^4^; *A. cupuliferum* Regel subsp. *cupuliferum* FM177292^6^; *A. cyaneum* Regel—**HQ690563**; Taibaishan, Shanxi, China; Li Q-Q 09080806; *A. cyathophorum* Bureau & Franch.—**GQ181093**; Zhongdian, Yunnan, China; Xu J-M 93-17; *A. decipiens* Fisch. ex Schult. & Schult.f. FM177306^6^; *A. drepanophyllum* Vved. AJ411854^4^; *A. drobovii* Vved. AJ411895^4^; *A. drummondii* Regel AJ411908^4^; *A. eduardii* Stearn ex Airy Shaw—**GQ181100**; Daqing Mountain, Nei Mongol, China; He X-J et al. 97829; *A. fascicμlatum* Rendle—**GQ181068**; Dazi, Xizang, China; Tu Y-L et al.94-9; *A. fetisowii* Regel FM177316^6^; *A. filidentiforme* Vved. AJ412722^4^; *A. flavescens* Besser AJ411842^4^; *A. flavovirens* Regel—**GQ181069**; Bayanhoti, Nei Mongol, China; He X-J *et al.* 97830; *A. forrestii* Diels—**GQ181070**; Queer Mountain, Sichuan, China; Zhang Y-C B24; *A. funckiifolium* Hand.-Mazz. 1—**HQ690557**; Wanrenkeng, Wushan, Chongqing; L Q-Q 2010071601; *A. galanthum* Kar. & Kir.—**GQ181101**; Altai, Xinjiang, China; He X-J & Zhang X-L 97631; *A. giganteum* Regel FM177320^6^; *A. glandulosum* Link & Otto AJ412746^4^; *A. goodingii* Ownbey AF055095^2^; *A. griffithianum* Boiss. AJ411862^4^; *A. gunibicum* Miscz. ex Grossh AM418361^5^; *A. gypsaceum* Popov & Vved. FM177322^6^; *A. haematochiton* S.Watson EU096157^7^; *A. hexaceras* Vved. FM177326^6^; *A. hookeri* Thwaites AJ412740^4^; *A. hookeri* var. *mμliense* Airy Shaw—**GQ181071**; Zhongdian ,Yunnan, China; Xu J-M 93 -25; *A. inaequale* Janka AJ412735^4^; *A. insubricum* Boiss. & Reut. AJ250291^3^; *A. insufficiens* Vved. FM177334^6^; *A. karataviense* Regel FM177341^6^; *A. koelzii* (Wendelbo) Perss. & Wendelbo FM177313^6^; *A. komarovianum* Vved. AJ412760^4^; *A. komarowii* Lipsky FM177342^6^; *A. kopetdagense* Vved. AJ411950^4^; *A. kuhsorkhense* R.M.Fritsch & Joharchi FM177386^6^; *A. kujukense* Vved. AJ411947^4^; *A. leucocephalum* Turcz. ex Ledeb. AJ412757^4^; *A. lineare* L. —**GQ181103**; Wμlumuqi, Xinjiang, China; He X-J & Zhang X-L 97606; *A. lipskyanum* Vved. FM177348^6^; *A. listera* Stearn 1 —**GQ181063**; Dongfeng, Jilin, China; Tuliguer 91001; *A. listera* Stearn 2— **HQ690558**; Xianwutaishan, Hebei China; Zhao L-H 10071904; *A. litvinovii* Drobow ex Vved. AJ412727^4^; *A. macranthum* Baker—**HQ690562**; Taibaishan, Shanxi, China; Li Q-Q 09080901; *A. macrostemon* Bunge—**GU566613**; Wenchuan, Sichuan, China; Li Q-Q 09060601; *A. mairei* H.Lévl. —**HQ690574**; Jingdong, Yunnan, China; Wang Z-X 2010091008; *A. maowenense* J. M.Xu—**GQ181073**; Wenchuan, Sichuan, China; Yu H 97836; *A. margaritae* B.Fedtsch. AJ412732^4^; *A. membranaceum* Ownbey ex Traub EU096165^7^; *A. microdictyon* Prokh. 1 GQ412216^8^; *A. microdictyon* Prokh. 2 AJ411859^4^; *A. minutiflorum* Regel FM177446^6^; *A. moly* L. AF055108^2^; *A. monanthum* Maxim. AJ412745^4^; *A. mongolicum* Regel—**GQ181074**; Saihan, Nei Mongol, China; He X-J *et al.* 97802; *A. nanodes* Airy Shaw 1—**GU566615**; Daocheng, Sichuan, China; Li Q-Q 09080101; *A. nanodes* Airy Shaw 2—**HQ690554**; Wumingshan, Xiangcheng, Sichuan; Li Q-Q 092302; *A. neriniflorum* (Herb.) G.Don —**HQ690560**；Songshan, Beijing, China; W Z-X 2010071506; *A. ochotense* Prokh.1 GQ412223^8^; *A. omeiense* Z.Y.Zhu—**HQ690567**; Emei Mountain, Sichuan, China; Hu H-Y em-20100626-2; *A. oreophilum* C.A.Mey. AF037620^1^; *A. oreoprasum* Schrenk—**GQ181105**; Tuoli, Xinjiang, China; He X-J & Zhang X-L 97619; *A. ovalifolium* var. *cordifolium* (J.M.Xu) J.M.Xu 1—**HQ690555**; Laokangmao, Shuajingsi, Hong yuan, Sichuan, China; Li Q-Q & Wei X-Q 2009061002; *A.ovalifolium* var. *leuconeurum* (J.M.Xu) J.M.Xu—**GU566616**; Dabanzhao, Lixian, Sichuan, China; Li Q-Q 09060901; *A.ovalifolium* var. *leuconeurum* (J.M.Xu) J.M.Xu 2— **KP207687**; Shanjiaoba, Lixian, Sichuan, China; Li Q-Q & Wei X-Q 2009060905; *A. ovalifolium* Hand.-Mazz. var. *ovalifolium* 1—**HQ690520**; Dabanzhao, Lixian, Sichuan, China; Li Q-Q & Wei X-Q 2009060802; *A. ovalifolium* Hand.-Mazz. var. *ovalifolium* 2—**HQ690521**; Donggouxipo, Shua jing si, Hong yuan, Sichuan, China; Li Q-Q & Wei X-Q 2009061003; *A. ovalifolium* Hand.-Mazz. var. *ovalifolium* 3—**HQ690522**; Huishuigou, Fubianxiang, Xiaojin, Sichuan, China; Li Q-Q & Wei X-Q 2009061301; *A. ovalifolium* Hand.-Mazz. var. *ovalifolium* 4—**HQ690526**; Xinglongshan, Yuzhong, Gansu, China; Wei X-Q 2009072507; *A. ovalifolium* Hand.-Mazz. var. *ovalifolium* 6—**HQ690528**; Huanglong, Songpan, Sichuan, China; Ma X-G C09091901; *A. pallasii* Murr. —**GQ181077**; Emin, Xinjiang, China; He X-J & Zhang X-L 97603; *A. parvulum* Vved. AJ412720^4^; *A. plurifoliatum* Rendle var. *plurifoliatum*—**GU566617**; Taibaishan, Shanxi, China; Li Q-Q 09080807; *A. polyrhizum* Turcz. ex Regel—**GQ181107**; Saihan, Nei Mongol, China; He X-J *et al.* 97804; *A. praecox* Brandegee EU096173^7^; *A. prattii* C.H.Wright ex Hemsley 1—**HQ690523**; Sandaoqiao, Kangding, Sichuan, China; Li Q-Q & Wei X-Q2009062701; *A. prattii* C.H.Wright ex Hemsley 2—**HQ690524**; Lushan, Xichang, Sichuan, China; Gao Y-D G20090726-5; *A. prattii* C.H.Wright ex Hemsley 3—**HQ690525**; Shuduhu, Xianggelila, Yunan, China; Li Q-Q BT09072803; *A. prattii* C.H.Wright ex Hemsley 5—**GQ181087**; Xiaojin, Sichuan, China; Li Q-Q 2008081603; *A. prattii* C.H.Wright ex Hemsley 7—**HQ690533**; Wengongmiao, Taibaishan, Shanxi, China; Li Q-Q 09080903; *A. prattii* C.H.Wright ex Hemsley 10—**HQ690537**; Bowashan, Daocheng, Sichuan, China; Li Q-Q BW09080102; *A. prattii* C.H.Wright ex Hemsley 17—**HQ690544**; Yakou, Changdu to Leiwuqi, Tibet, China; Gao Y-D 10-1237; *A. prattii* C.H.Wright ex Hemsley 22—**HQ690542**; Awangxiang to Dongshaxiang, Gongjue, Tibet, China; Gao Y-D 10-1918; *A. prattii* C.H.Wright ex Hemsley 27—**HQ690552**; Daxueshanyakou, Xianggelila, Yunnan, China; Gao Y-D 20100718; *A. przewalskianum* Regel—**HQ690568**; Daluoshan, Ningxia, China; Wang C-B 09098; *A. pseudobodeanum* R.M.Fritsch & Matin FM177381^6^; *A. pseudowinklerianum* R.M.Fritsch & F.O.Khass. FM177387^6^; *A. punctum* L.F.Hend. EU096174^7^; *A. ramosum* L. —**HQ690285**; Manzhouli, Nei Mongol, China; Wang C-B 09068; *A. regelii* Trautv. FM177389^6^; *A. rosenorum* R.M.Fritsch FM177395^6^; *A. rude* J.M.Xu—**HQ690561**; Litang, Sichuan, China; Li Q-Q 092202; *A. sarawschanicum* Regel FM177406^6^; *A. sativum* L. AF037621^1^; *A. saxatile* M..Bieb. —**GQ181108**; Wμlumuqi, Xinjiang, China; He X-J & Zhang X-L 97626; A. *scabriscapum* Boiss. AJ411881^4^; *A. schachimardanicum* Vved. FM177410^6^; *A. schoenoprasoides* Regel AJ412728^4^; *A. schubertii* Zucc. FM177411^6^; *A. semenovii* Regel AJ411897^4^; *A. senescens* L. —**HQ690569**; Yichun, Heilongjiang, China; Wang C-B 09053; *A. sergii* Vved. AJ411936^4^; *A. sharsmithiae* (Ownbey & Aase) McNeal EU096179^7^; *A. shevockii* McNeal EU096180^7^; *A. siculum* Ucria AJ250299^3^; *A. siskiyouense* Munz & Keck ex Ownbey EU096181^7^; *A. spicatum* (Prain) N.Friesen —**JX017613**; Rikaze, Xizang, China; Huang D-Q XZ2011082609; *A. suworowii* Regel FM177430^6^; *A. taeniopetalum* Popov & Vved. var. *taeniopetalum* FM177433^6^; *A. tanguticum* Regel—**GQ181089**; Zuodao, Qinhai, China; Feng T *et al.* 20080701; *A. tenuissimum* L. — **GQ181090**; Helingeer, Nei Mongol, China; Li Q-Q 20080902; *A. tricoccum* Sol. AJ411917^4^; *A. triquetrum* L. AJ412742^4^; *A. tuberosum* Rottler ex Spreng. —**HQ690565**; Wenchuan, Sichuan, China; Li Q-Q & Wei X-Q 2009060502; *A. tubiflorum* Rendle—**GU566622**; Hua Mountain, Shanxi, China; Liao C-Y C072804; *A. umbilicatum* Boiss. AJ412719^4^; *A. ursinum* L. AJ412744^4^; *A. validum* S.Watson EU096188^7^; *A. verticillatum* Regel FM177447^6^; *A. victorialis* L. 1—**GQ181082**; Dunhua, Jilin, China; Tuliguer 91002; *A. victorialis* L. 2 AJ411919^4^; *A. viridulum* Ledeb. FM177449^6^; *A. wallichii* Kunth var. *wallichii*—**HQ690566**; Yulongxueshan, Yunnan, China; Li Q-Q YL09072630; *A. wallichii* var. *platyphyllum* (Diels) J.M.Xu—**GU566624**; Lijiang, Yunnan, China; Li Q-Q 09072503; *A. xichuanense* J.M.Xu— **HQ690564**; Zhegushan, Sichuan, China; Li Q-Q 2008081901; *A. yuanum* F.T.Wang & Tang —**GQ181092**; Maerkang, Sichuan, China; Li Q-Q 2008082003; *A. zergericum* F.O.Khass. & R.M.Fritsch FM177456^6^.

Outgroups: *Nothoscordum gracile* (Ait.) Stearn AJ412716^4^; Tulbaghia *violacea* Harv. — **GU566623**; Chengdu Botanical Garden, China; Li Q-Q *et al.* 091110.
